# Circulating gut microbial metabolites and risk of coronary heart disease: A prospective multi-stage metabolomics study

**DOI:** 10.1371/journal.pmed.1004750

**Published:** 2026-03-17

**Authors:** Yulu Zheng, Jae Jeong Yang, Deepak K. Gupta, David M. Herrington, Bing Yu, Ngoc Quynh H. Nguyen, Rui Pinto, Ioanna Tzoulaki, Hui Cai, Qiuyin Cai, Loren Lipworth, Xiao-Ou Shu, Wei Zheng, Danxia Yu

**Affiliations:** 1 Division of Epidemiology, Department of Medicine, Vanderbilt University Medical Center, Nashville, Tennessee, United States of America; 2 UF Health Cancer Center, Department of Surgery, College of Medicine, University of Florida, GainesvilleFlorida, United States of America; 3 Division of Cardiovascular Medicine, Department of Medicine, Vanderbilt University Medical Center, Nashville, Tennessee, United States of America; 4 Section on Cardiology, Department of Internal Medicine, Wake Forest School of Medicine, Winston-Salem, North Carolina, United States of America; 5 Department of Epidemiology and Human Genetics Center, UTHealth School of Public Health, Houston, Texas, United States of America; 6 Department of Epidemiology and Biostatistics, School of Public Health, Imperial College London, London, United Kingdom; University of Calgary, CANADA

## Abstract

**Background:**

Despite growing evidence linking gut microbiota and microbial metabolites to human cardiometabolic health, few studies have systematically examined associations between circulating microbial metabolites and incident coronary heart disease (CHD).

**Methods and findings:**

We conducted a multi-stage metabolomics study involving five prospective cohorts. Discovery involved untargeted plasma metabolite profiling of 896 incident cases and 896 age-/sex-/race-matched controls (~300 pairs per race: Black, White, Asian) from the Southern Community Cohort Study (SCCS; baseline: 2002–2009) and the Shanghai Women’s Health Study and Shanghai Men’s Health Study (SWHS/SMHS; baseline: 1996–2000 and 2002–2006). In-silico validation was conducted in the Atherosclerosis Risk in Communities Study (ARIC; *N* = 3,539; 663 cases; baseline: 1987–1989) and Multi-Ethnic Study of Atherosclerosis (MESA; *N* = 3,860; 446 cases; baseline: 2000–2002). Lastly, a quantitative assay was developed and applied to a new set of 864 cases and 864 age-/sex-/race-matched controls (~260−340 pairs per race) from the SCCS and SWHS/SMHS. Conditional logistic regression estimated odds ratios (ORs) of incident CHD per standard deviation (SD) metabolite increase in discovery and quantitative stages with a nested case-control design. Cox regression was used in ARIC and MESA with a cohort design. Similar covariates were adjusted across stages, including age, sex (if applicable), race (if applicable), education, income, smoking status, alcohol consumption, physical activity, diet quality, and body mass index (BMI). The mean (SD) time between enrollment and CHD diagnosis was 5.6 (3.8), 6.9 (4.4), 15.0 (7.4), and 8.0 (4.9) years in the SCCS, SWHS/SMHS, ARIC, and MESA, respectively. The discovery stage identified 73 circulating microbiota-related metabolites associated with incident CHD (false discovery rate <0.10). Sixty-one metabolites were available for in-silico validation, of which 24 showed a significant association (*p* < 0.05) in the same direction as in the discovery. The targeted assay quantified eight of the 24 metabolites, with five significantly associated with incident CHD: imidazole propionate, 3-hydroxy-2-ethylpropionate, 4-hydroxyphenylacetate, *trans-*4-hydroxyproline, and 3-hydroxybutyrate; OR per SD ranged from 1.18 to 1.27 after adjustment for sociodemographics, lifestyles, and BMI. The targeted assay measured eight other promising microbial metabolites, four of which were significant: trimethylamine N-oxide, phenylacetyl-L-glutamine, 4-hydroxyhippuric acid, and indolepropionate. Most associations were consistent across participant subgroups by demographics, lifestyles, metabolic disease history, family CHD history, and follow-up time, although some potential effect modifications were found by race, age, obesity status, and follow-up time. The main limitations of the study are the observational design and the inability to validate all significant metabolites due to differences in metabolomic assay coverage across the three stages.

**Conclusions:**

We identified and validated circulating gut microbial metabolites associated with incident CHD across diverse populations. Our findings offer novel epidemiological evidence on the importance of gut microbial metabolism in CHD development and highlight specific metabolites to prioritize for mechanistic investigation, biomarker validation, and therapeutic development.

## Author summary

**Why was this study done?:**

- Gut microbiota affects human cardiovascular health, including the risk of coronary heart disease.
- Metabolomics technology has been used to examine the role of microbiota-related metabolites in the development of cardiovascular diseases.
- Evidence from prospective studies with rigorous discovery and validation stages across racially and geographically diverse populations remains limited.

**What did the researchers do and find?:**

- We conducted a multi-stage metabolomic study to evaluate blood levels of microbiota-related metabolites in association with risk of developing coronary heart disease among Black, White, and Asian adults.
- We identified and validated nine circulating microbial metabolites linked to risk of coronary heart disease across diverse populations, including metabolites of amino acids, lipids, and xenobiotics.

**What do these findings mean?:**

- Our findings underscore the importance of gut microbiota and microbial metabolism in cardiovascular disease development and highlight promising metabolites that may serve as novel biomarkers or therapeutic targets for future mechanistic and interventional studies.
- The main limitation of this study is its observational design, which means we can identify metabolites associated with disease risk but cannot prove that metabolites caused the development of the disease.
- Another limitation of this study is that we cannot confirm all potentially important metabolites due to differences in metabolomic assays across stages.

## Abstract 3

In this metabolomics study, Yulu Zheng and colleagues investigate associations between microbial metabolites and coronary heart disease, performing discovery and validation analyses using five different cohorts.

## Introduction

Coronary heart disease (CHD) remains the leading cause of death in the United States (US) and worldwide, with incidence varying by race/ethnicity, socioeconomic status, and geographic area [1,2]. Emerging research on gut microbiota has provided insights into the etiology and prevention of CHD, with potential implications for the development of new therapeutics [3–6]. With a genome 100 times larger than humans’, gut microbiota generates numerous small molecules (metabolites), many of which humans cannot produce. Microbial metabolites can enter the host circulation and exert systemic, multifaceted effects on human health and disease, including cardiovascular, metabolic, inflammatory, and neurological disorders [7–10]. Prominent examples of gut microbial metabolites include short-chain fatty acids from fiber fermentation, trimethylamine N-oxide (TMAO) from phosphatidylcholine and l-carnitine, secondary bile acids from cholesterol and primary bile acids, and amino acid metabolites such as indoles from tryptophan, imidazole propionate from histidine, and phenylacetylglutamine from aromatic amino acids (AroAA).

While metabolomics has been increasingly applied to cardiovascular disease (CVD) research [11–14], evidence from human studies linking microbial metabolites to CHD or CVD was mainly from cross-sectional studies or clinical patient cohorts, prone to reverse causation and confounding bias. Additionally, most prior studies examined a small number of selected microbial metabolites. Comprehensive investigations of microbial metabolites in population-based, prospective studies with rigorous validation of findings are crucial. Further, few prospective studies examined microbial metabolites in populations with high CVD burden [15,16], such as Black Americans and low-income individuals. For gut microbiota-related research, data from populations with varying sociodemographic, geographic, and diet/lifestyle backgrounds are highly valuable. Studies have shown distinct metabolomic profiles among individuals with different diets/lifestyles, with many distinguishing metabolites related to gut microbiota [17–19]. Whether microbial metabolites contribute to CHD risk across diverse populations warrants investigation.

Hence, we conducted a multi-stage metabolomic study involving demographically and geographically diverse participants, including: (1) an untargeted, semi-quantitative assay for discovery among 896 incident CHD cases and 896 age-/sex-/race-matched controls (~300 pairs per race: Black, White, and Asian/Chinese) from the Southern Community Cohort Study (SCCS) and Shanghai Women’s Health Study and Shanghai Men’s Health Study (SWHS/SMHS); (2) an in-silico validation in the Atherosclerosis Risk in Communities Study (ARIC) and Multi-Ethnic Study of Atherosclerosis (MESA); (3) a targeted, quantitative assay to measure metabolite concentrations, verify their associations with CHD, and evaluate potential microbiota-host interactions in a new set of 864 incident CHD cases and 864 matched controls (~260–340 pairs per race) from SCCS and SWHS/SMHS. This study aimed to enhance understanding of the role of gut microbial metabolites in CHD development and to inform potential novel biomarkers or prevention strategies.

## Methods

### Study population and design

This study had a prospective protocol. Detailed descriptions of the study designs and protocols for SCCS, SWHS, SMHS, ARIC, and MESA were published elsewhere [20–25]. Briefly, all are population-based prospective cohorts that recruited participants, conducted surveys, collected biospecimens including peripheral blood, and followed participants for disease outcomes including incident CHD and CHD death. SCCS enrolled ~85,000 participants (40–79 years) from 12 southern US states in 2002–2009, mostly Black/African American adults with low household incomes [20]. SWHS enrolled ~75,000 women (40–70 years), and SMHS enrolled ~61,000 men (40–74 years) from Shanghai, China, in 1996–2000 and 2002–2006, respectively [21,22]. ARIC enrolled ~16,000 participants (45–65 years) from four US centers in 1987–1989, with ~27% being Black/African American [23,24]. MESA enrolled 6,814 participants (45–84 years) from six US centers in 2000–2002, with ~38% being White, 28% Black/African American, 22% Hispanic/Latino, and 12% Chinese [25]. See Protocol in S1 Text for detailed information. All participants in SCCS, SWHS, SMHS, ARIC, and MESA provided written informed consent at enrollment. All cohorts received IRB approval from the participating institutions. The Vanderbilt University Medical Center IRB approved the present study (#201082). This study is reported as per the Strengthening the Reporting of Observational Studies in Epidemiology (STROBE) guideline (S1 STROBE Checklist).

For discovery and targeted validation, prospective nested case-control studies were conducted in SCCS and SWHS/SMHS. Participant inclusion criteria were (1) no history of CHD, stroke, heart failure, cancer, or end-stage renal disease at baseline; (2) available plasma samples and data on fasting time, and in SCCS, the time between sample collection and lab processing; (3) no use of antibiotics nor cold/flu in the last seven days before blood collection to minimize the potential influence of acute disease and medications on gut microbiota; (4) in SCCS, participants were covered by the Centers for Medicare and Medicaid Services (CMS) and had ≥2 claims after cohort enrollment through December 2016 to identify those with continued CMS coverage and ensure the validity of claims data; in SWHS/SMHS, participants’ medical records were accessible for this study through December 2016. In SCCS, nonfatal CHD cases were identified through CMS data, including acute myocardial infarction (MI) and coronary revascularization, and CHD deaths were identified through the National Death Index. In SWHS/SMHS, CHD cases were initially identified by self-reports of physicians' diagnoses during follow-up visits and confirmed by medical records. CHD cases were 1:1 matched with controls without a history of CVD or cancer at the time of case diagnosis using incidence density sampling by race, sex, enrollment age (±2 years), fasting time (±2 hours), and time between blood collection and lab processing (±4 hours for SCCS; all SWHS/SMHS samples were processed within 6 hours after collection). After applying inclusion and case-control matching criteria, 150 case-control pairs in each race (Black, White, Asian) and sex (male, female) were randomly selected for discovery, with the remainder included for targeted validation: Black women (167 pairs), Black men (170 pairs), White women (149 pairs), White men (109 pairs), Chinese women (120 pairs), and Chinese men (148 pairs). The sample selection was conducted in August 2020.

The in-silico validation was conducted in ARIC and MESA using a prospective cohort design. In ARIC, 3,539 participants with plasma metabolite data at baseline were included after excluding those with a history of CHD, heart failure, stroke, or cancer. Incident CHD was defined as definite or probable MI, fatal CHD, and if participants had undergone any cardiac procedures or ECG MI before December 31, 2018. In MESA, 3,860 participants with baseline plasma metabolite data were included after excluding participants with a history of heart disease or stroke, or who were not fasting. Incident CHD was defined as MI, resuscitated cardiac arrest, and CHD death occurring on or before and adjudicated through December 31, 2018.

### Metabolites profiling

For discovery and targeted validation, baseline plasma samples from case-control pairs were retrieved and placed adjacent to each other in the same batch at random. Laboratory personnel were blinded to the case-control status. For discovery, untargeted metabolite profiling was performed using ultra-high-performance liquid chromatography with tandem mass spectrometry (UHPLC–MS/MS) in positive and negative ion modes using a combination of reverse phase and HILIC chromatography methods by Metabolon from May to August 2021. The general assay protocol has been published [26]. A brief description is provided in Protocol in S1 Text. Overall, plasma samples were extracted with methanol and split into four aliquots for UHPLC–MS/MS assays in both positive and negative ion modes using a combination of reverse phase and HILIC chromatography methods. Metabolites were identified by comparing mass spectral features to a reference library of >5,000 authenticated standard compounds. A total of 1,503 metabolites were detected in our discovery stage samples. For the present study, we focused on 226 gut microbiota-related metabolites identified by two methods: (1) linkage to a database of 460 confirmed gut microbial metabolites via Exposome-Explorer [27]; (2) regression analysis of metabolite levels (dependent variable) with oral antibiotic use seven days before blood collection (independent variable) to identify metabolites significantly reduced by recent antibiotic use using previous data from the SWHS/SMHS (1,841 participants; 1,498 metabolites measured by the same Metabolon panel [28]). These two methods identified 165 and 74 microbiota-related metabolites (13 overlapped), respectively. The median coefficient of variation (CV) for those 226 metabolites among our QC samples was 9.7% (interquartile range: 5.6%−16.3%).

In ARIC, baseline fasting serum samples were analyzed by the same Metabolon panel using UHPLC–MS/MS, which detected 787 metabolites [26]. In MESA, baseline samples were analyzed using nuclear magnetic resonance and LC–MS, with detailed methods previously published [29–31]. A brief description is provided in Protocol in S1 Text. Metabolites were harmonized using HMDB ID and PubChem ID across cohorts.

For targeted validation, metabolite concentrations were quantified using LC–MS/MS by Metabolon from June 2023 to February 2024. Specifically, plasma samples were spiked with isotopically labeled internal standards and subjected to protein precipitation with an acidified organic solution. An aliquot was injected onto an Agilent 1290/Sciex Triple Quad 6500+ LC–MS/MS system equipped with either an Agilent Zorbax SB-C18 RRHD for the reverse phase method or a Waters Acquity Premier BEH Amide column for the HILIC method. The mass spectrometer was operated in both positive and negative modes using heated electrospray ionization. The peak area of the individual analyte product ions was measured against the peak area of the product ions of the corresponding internal standards. Quantitation was performed using a weighted linear least squares regression analysis generated from fortified calibration standards prepared concurrently with study samples. Sample analysis was conducted in a 96-well plate format containing two calibration curves and six QC samples (three levels with two at each). Accuracy was evaluated using the QC replicates in the sample runs. The average accuracy for QC at all levels for all 16 analytes was >90% except for 1-methyl-4-imidazoleacetate (88%). The median CV for the 16 metabolites among our study QC samples was 3.6% (interquartile range: 1.6%−6.4%). Detailed information on reference internal standards, calibration ranges, and QC performance can be found in Protocol in S1 Text.

For discovery and in-silico validation stages using untargeted metabolomics, metabolite levels below the detection limit were imputed using the minimum value among non-missing samples within each study and each batch. Given the large sample size, we included metabolites with up to 60% missingness; however, metabolites with higher missingness were less likely to advance to the validation stage. For quantitative validation, undetectable metabolites were treated as missing (12 of the 16 metabolites had none or <1% missing; 3 metabolites had 5%–10% missing; 1 metabolite [4-hydroxyphenylpyruvate] had 23% missing). Metabolite levels were all log-transformed and z-scored within each study and each batch prior to statistical analyses.

## Statistical analysis

**Fig 1:**
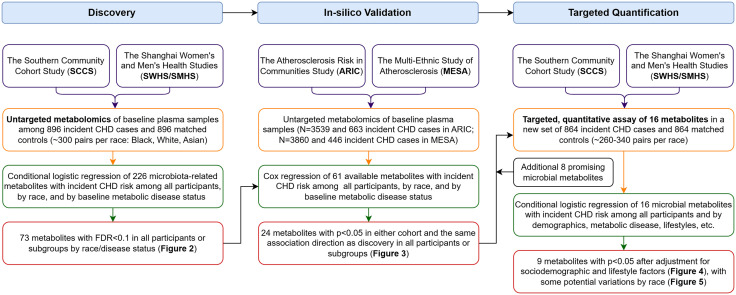
Study flowchart.

In discovery and targeted validation stages with a matched case-control design, conditional logistic regression was applied to compute odds ratios (ORs) and 95% confidence intervals (95% CIs) for a standard deviation (SD) increase in log-transformed metabolites among all participants and by race. Model 1 adjusted for age (continuous); Model 2 additionally adjusted for educational attainment (less than high school, high school graduation, vocational school, college or more), income (cohort-specific low, middle, and high), smoking status (never, former, current with <20 cigarettes/day, current with ≥20 cigarettes/day), alcohol consumption (none, moderate [≤2 drinks/day for men, ≤1 drink/day for women], heavy), physical activity (cohort-specific tertiles), diet quality score (cohort-specific tertiles; specifically, Healthy Eating Index per *Dietary Guidelines for Americans* in the US cohorts and Chinese Food Pagoda Score per *Dietary Guidelines for Chinese* in the SWHS/SMHS, as described in our previous publications [32,33]), and BMI (continuous). We also conducted stratified analyses by baseline status of diabetes, hypertension, and dyslipidemia using logistic regression with additional adjustment for matching variables (sex, race, fasting status). Disease status was defined by self-reports of doctors’ diagnoses or medication usage to treat the disease. Metabolites showing Benjamini–Hochberg false discovery rate (FDR) adjusted *p*-values < 0.1 in either model among all participants or in any subgroups by race/disease status entered in-silico validation. The in-silico validation was conducted in ARIC and MESA using Cox regression with the same covariates described above, plus center and batch. The analyses were performed within each cohort among all eligible participants and by race and by baseline diabetes, hypertension, and dyslipidemia. Metabolites were considered validated if nominal *p* < 0.05 in any cohort with the same direction of association. Statistical analyses for discovery, in-silico validation, and targeted validation were conducted from September to December 2021, from February to May 2022, and from April to October 2024, respectively, with covariates and significance levels determined a priori. SAS Enterprise (SAS Institute, Cary, North Carolina, US) and R (version 4.3.3) were used for analysis. A flowchart showing the study design, methods, and results across stages is shown in Fig 1.

## Results

### Baseline characteristics of study participants

Mean age at baseline was ~57 years (SD: 9) for included SCCS and SWHS/SMHS participants (Table 1). Compared to age/sex/race-matched controls, individuals who developed incident CHD over a mean follow-up of ~6 years (SD: 4) had lower levels of education and household income and were more likely to be current smokers, non-drinkers, and have low leisure-time physical activity. In addition, CHD cases showed higher baseline BMI and greater prevalence of diabetes, hypertension, dyslipidemia, and family history of CHD. Characteristics of participants included in in-silico validation are shown in S1 Table. Briefly, ARIC participants had a mean age of ~54 years at visit 1, with 60.3% being women, 38.5% White, and 61.5% Black/African American. MESA participants had a mean age of ~63 years at baseline, with 50.7% being women, 39.1% non-Hispanic White, 23.6% Black/African American, 13.7% Chinese American, and 23.7% Hispanic. The mean (SD) time between baseline and CHD diagnosis was 15.0 (7.4) and 8.0 (4.9) years in ARIC and MESA, respectively.

**Table 1:** Baseline characteristics of participants in the discovery and targeted validation stages.

	Discovery stage		Targeted validation stage	
	CHD cases (*N* = 896)	Matched controls (*N* = 896)	CHD cases (*N* = 864)	Matched controls (*N* = 864)
**Age, years**	57.2 (9.1)	57.1 (9.0)	56.6 (9.0)	56.6 (8.9)
**Female, *n* (%)**	450 (50.2)	450 (50.2)	437 (50.6)	437 (50.6)
**Race, *n* (%)**				
Black/African American	298 (33.3)	298 (33.3)	259 (30.0)	259 (30.0)
Non-Hispanic White	299 (33.4)	299 (33.4)	337 (39.0)	337 (39.0)
Asian	299 (33.4)	299 (33.4)	268 (31.0)	268 (31.0)
**Education, *n* (%)**				
Less than high school	409 (45.6)	377 (42.1)	416 (48.1)	317 (36.7)
Completed high school	292 (32.6)	287 (32.0)	233 (27.0)	298 (34.5)
Vocational school or some college	138 (15.4)	136 (15.2)	149 (17.2)	154 (17.8)
College or graduate school	57 (6.4)	96 (10.7)	66 (7.6)	95 (11.0)
**Income** ^1^ **, *n* (%)**				
Low	449 (50.1)	406 (45.3)	425 (49.2)	394 (45.6)
Middle	416 (46.4)	446 (49.8)	347 (40.2)	333 (38.5)
High	31 (3.5)	44 (4.9)	92 (10.6)	137 (15.9)
**Smoking status** ^2^				
Never	352 (39.3)	413 (46.1)	310 (35.9)	390 (45.1)
Former	194 (21.6)	176 (19.6)	168 (19.4)	181 (20.9)
Current, cigarettes per day < 20	186 (20.8)	169 (18.9)	260 (30.1)	199 (23.0)
Current, cigarettes per day ≥ 20	164 (18.3)	138 (15.4)	126 (14.6)	94 (10.9)
**Alcohol intake** ^3^ **, *n* (%)**				
None	587 (65.5)	535 (59.7)	541 (62.6)	492 (56.9)
Moderate	214 (23.9)	267 (29.8)	212 (24.5)	252 (29.2)
Heavy	95 (10.6)	94 (10.5)	111 (12.8)	120 (13.9)
**Diet quality** ^4^ **, *n* (%)**				
Low	308 (34.4)	265 (29.6)	295 (34.1)	275 (31.8)
Middle	318 (35.5)	328 (36.6)	276 (31.9)	294 (34.0)
High	270 (30.1)	303 (33.8)	293 (33.9)	295 (34.1)
**Leisure-time physical activity** ^5^ **, *n* (%)**				
Low	309 (34.5)	278 (31.0)	313 (36.2)	257 (29.7)
Middle	289 (32.3)	332 (37.1)	273 (31.6)	297 (34.4)
High	298 (33.3)	286 (31.9)	278 (32.2)	310 (35.9)
**Body mass index, kg/m** ^**2**^	29.0 (6.9)	27.9 (6.6)	29.0 (6.5)	28.0 (6.6)
**Family history of CHD** ^6^ **, *n* (%)**	318 (35.5)	264 (29.5)	301 (34.8)	245 (28.4)
**History of diabetes** ^7^ **, *n* (%)**	270 (30.1)	121 (13.5)	249 (28.8)	120 (13.9)
**History of hypertension** ^7^ **, *n* (%)**	538 (60.0)	413 (46.1)	568 (65.7)	388 (44.9)
**History of dyslipidemia** ^7^ **, *n* (%)**	291 (32.5)	221 (24.7)	364 (42.1)	249 (28.8)
**Blood triglycerides, mg/dL**	166.5 (146.1)	142.3 (125.8)	149.7 (128.0)	122.8 (102.6)
**Blood total cholesterol, mg/dL**	244.3 (72.4)	236.6 (66.0)	213.1 (74.2)	201.8 (62.8)
**LDL cholesterol, mg/dL**	123.5 (54.2)	120.0 (48.7)	105.9 (50.4)	100.3 (44.5)
**HDL cholesterol, mg/dL**	56.5 (18.8)	59.5 (20.4)	49.8 (15.8)	52.4 (18.2)
**Time between enrollment and CHD diagnosis, years**	6.2 (4.1)	–	5.6 (3.8)	–

Cases and controls were 1:1 matched using incidence density sampling by sex, race, enrollment age (±2 years), fasting time, and time between blood draw and lab processing. Data were presented as mean (standard deviation) for continuous variables with normal distributions (age, BMI), median (interquartile range) for continuous variables with non-normal distributions (blood lipids), and *n* (%) for categorical variables.

^1^Low, middle, and high levels of income were defined by annual household income < $15,000, $15,000 to <$25,000, and ≥$25,000 in the SCCS, annual household income <¥4,000, ¥4,000 to <¥8,000, and ≥¥8,000 in the SWHS, and annual personal income <¥6,000, ¥6,000 to <¥10,000, and ≥¥10,000 in the SMHS.

^2^In SCCS, never smokers were defined as someone who had not smoked ≥100 cigarettes in their entire life. Former smokers were defined as someone who had ever smoked ≥100 cigarettes but did not smoke at the baseline survey. In SWMHS, never smokers were defined as someone who had not smoked at least one cigarette per day for ≥6 months continuously. Former smokers were defined as someone who had ever smoked at least one cigarette per day for ≥6 months continuously but did not smoke regularly at the baseline survey.

^3^Moderate alcohol drinking was defined as >0 to ≤2 drinks per day in men or >0 to ≤1 drink per day in women, and heavy drinking was defined as >2 drinks per day in men or >1 drink per day in women (1 drink = 14 g ethanol).

^4^Diet quality was categorized based on tertiles of the Healthy Eating Index-2010 in the SCCS and the Chinese Food Pagoda Score in the SWHS/SMHS.

^5^Physical activity was categorized based on tertiles of the metabolic equivalent of task hours per week.

^6^Family history refers to the first degree of relatedness.

^7^Histories of diabetes, dyslipidemia, and hypertension were determined by self-reported doctor diagnosis or medication use.

CHD, coronary heart disease; LDL, low-density lipoprotein; HDL, high-density lipoprotein.

### Discovery of circulating microbiota-related metabolites associated with incident CHD

**Fig 2:**
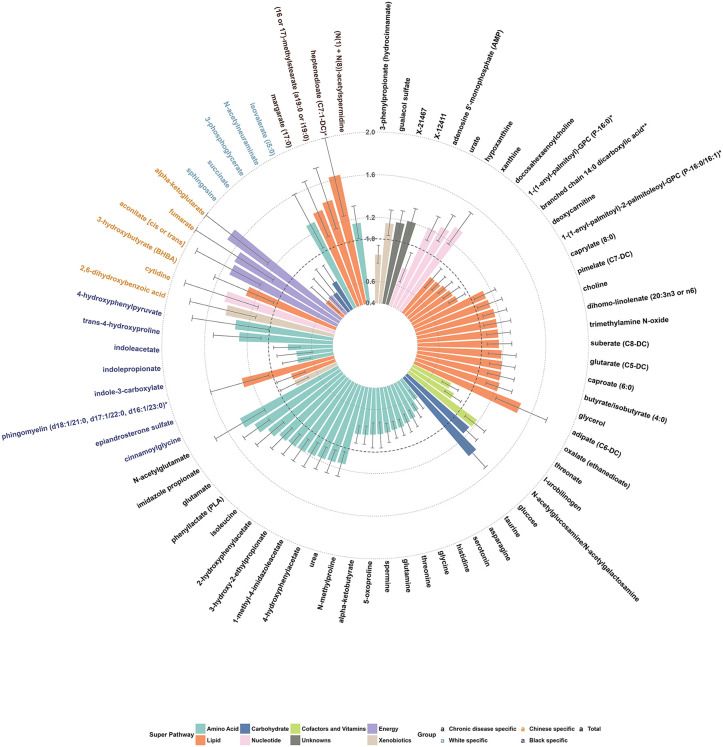
Discovery of circulating gut microbiota-related metabolites associated with incident coronary heart disease. The discovery stage applied untargeted, semi-quantitative metabolite profiling among 896 incident cases and 896 matched controls from SCCS and SWHS/SMHS. ORs and 95% CIs for per SD increase in log-transformed metabolite levels were obtained using conditional logistic regression among all participants and by race, with adjustment for age (model 1) or additionally, education, income, cigarette smoking, alcohol consumption, physical activity, diet quality score, and BMI (model 2). When stratified by metabolic disease history, logistic regression was used with additional adjustment for matching variables (sex, race, and fasting status). Metabolites showing FDR-*p* < 0.1 among total participants (black font) or in participant subgroups (Black: purple font; White: teal font; Asian: orange font; by disease: brown) are presented (see S2 Table for more details).

In the discovery stage, 48 of 226 microbiota-related metabolites showed a significant association with incident CHD (FDR < 0.1) in Model 1. All but five metabolites remained significant after further adjustments for sociodemographics, lifestyles, BMI, and prevalent metabolic diseases (FDR < 0.1 in Model 2); meanwhile, two metabolites became significant after additional adjustments. Furthermore, 23 metabolites were significantly associated with incident CHD in participant subgroups, including 19 in race-specific analyses and 4 in stratified analyses by metabolic disease status. A total of 73 metabolites were significant in either model among all participants or subgroups by race/disease status. They were from super pathways of amino acids (*n* = 28), lipids (*n* = 23), nucleotides (*n* = 5), carbohydrates (*n* = 4), energy (*n* = 4), xenobiotics (*n* = 4), cofactors/vitamins (*n* = 3), and unknowns (*n* = 2), with ORs per SD ranging from 0.61 to 0.88 for inverse associations and 1.13 to 1.71 for positive associations (Fig 2). Detailed information (e.g., sub-pathway, HMDB ID, PubChem ID, and missing rate) and ORs (95% CI) for those metabolites in both models in total participants and subgroups are all shown in S2 Table.

### In-silico validation of circulating microbiota-related metabolites associated with incident CHD

**Fig 3:**
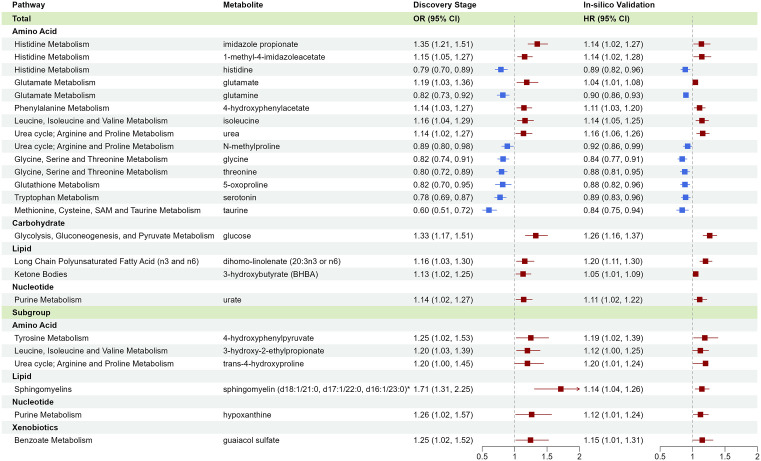
In-silico validation of circulating gut microbiota-related metabolites associated with incident coronary heart disease. The in-silico validation stage was conducted using existing untargeted, semi-quantitative metabolite data among 3,539 participants in ARIC (663 cases) and 3,860 participants in MESA (446 cases). HRs and 95% CIs for a per-SD increase in log-transformed metabolite levels were estimated using Cox regression among all participants and by race or metabolic disease history, with adjustments for sociodemographics, lifestyles, and BMI. Metabolites showing *p* < 0.05 (from Cox regression) in the same direction as the discovery results among total participants or participant subgroups are presented.

Among the 73 metabolites, 61 were available in ARIC or MESA, and 24 showed a significant association (*p* < 0.05 in either cohort) in the same direction as discovery (Fig 3). Most validated metabolites were from super pathways of amino acids (*n* = 17), including metabolites of histidine, glutamate, phenylalanine, tyrosine, isoleucine, arginine and proline, glycine, and tryptophan. Others included lipids and xenobiotics. Detailed results for all 61 metabolites are shown in S3 Table.

### Targeted validation of circulating microbiota-related metabolites associated with incident CHD

**Fig 4:**
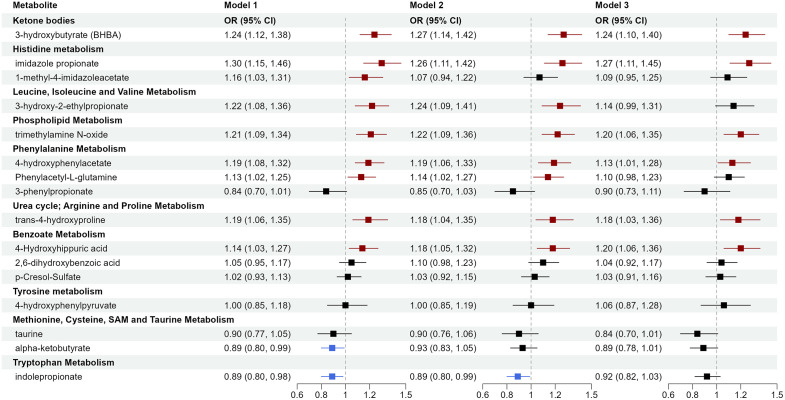
Targeted validation of circulating gut microbiota-related metabolites associated with incident coronary heart disease. The targeted validation stage applied quantitative profiling and measured 16 metabolites among 864 incident cases and 864 matched controls from SCCS and SWHS/SMHS. ORs and 95% CIs for per SD increase in log-transformed metabolite levels were obtained using conditional logistic regression among all participants. Model 1 adjusted for age (continuous). Model 2 additionally adjusted for education, income, cigarette smoking, alcohol consumption, physical activity, diet quality score, and BMI (continuous). Model 3 additionally adjusted for history of diabetes, hypertension, and dyslipidemia.

A quantitative assay was designed to measure concentrations of the most promising metabolites, which captured eight of 24 metabolites that passed the in-silico validation, including seven amino acid metabolites (imidazole-propionate, 1-methyl-4-imidazoleacetate, 3-hydroxy-2-ethylpropionate, 4-hydroxyphenylacetate, *trans-*4-hydroxyproline, 4-hydroxyphenylpyruvate, taurine) and ketone body 3-hydroxybutyrate. Due to different LC-MS requirements, the remaining 16 metabolites could not be quantified simultaneously. However, the assay was able to measure eight other metabolites that were significant in the discovery and reported in recent population studies linking them to CHD/CVD but unavailable or insignificant in in-silico validation, including metabolites of amino acids (phenylacetyl-L-glutamine, alpha-ketobutyrate, indolepropionate), lipids (TMAO), and xenobiotics (3-phenylpropionate, 4-hydroxyhippuric acid, 2,6-dihydroxybenzoic acid, p-cresol-sulfate). Plasma concentrations of the 16 metabolites among cases and controls and distributions by race are shown in S4 Table and Fig A in S1 Text. Generally, metabolite concentrations were more comparable between Black and White Americans than between American and Asian individuals. Spearman correlations between the metabolites, adjusted for age, sex, race, and fasting status, are shown in Fig B in S1 Text; most metabolites showed weak to modest correlations. After adjusting for sociodemographics and lifestyles, nine metabolites were significant (Fig 4; *p* < 0.05 in Model 2), including 3-hydroxybutyrate (OR per SD increase 1.27, 95% CI [1.14, 1.42]; *p* < 0.001), imidazole-propionate (OR 1.26, 95% CI [1.11, 1.42]; *p* < 0.001), 3-hydroxy-2-ethylpropionate (OR 1.24, 95% CI [1.09, 1.41]; *p* < 0.001), TMAO (OR 1.22, 95% CI [1.09, 1.36]; *p* < 0.001), 4-hydroxyphenylacetate (OR 1.19, 95% CI [1.06, 1.33]; *p* = 0.003), phenylacetyl-L-glutamine (OR 1.14, 95% CI [1.02, 1.27]; *p* = 0.02), *trans-*4-hydroxyproline (OR 1.18, 95% CI [1.04, 1.35]; *p* = 0.01), 4-hydroxyhippuric acid (OR 1.18, 95% CI [1.05, 1.32]; *p* = 0.005), and indolepropionate (OR 0.89, 95% CI [0.80, 0.99]; *p* = 0.04). Most associations remained significant after further adjustment for baseline diabetes, hypertension, and dyslipidemia (Model 3), except for 3-hydroxy-2-ethylpropionate, phenylacetyl-L-glutamine, and indolepropionate, suggesting they may affect incident CHD through those conditions.

**Fig 5:**
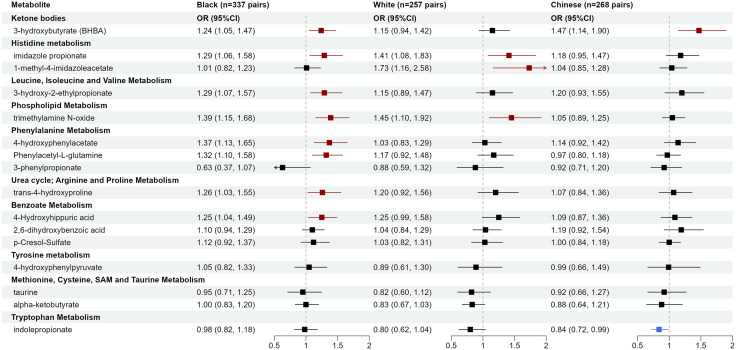
Circulating gut microbiota-related metabolites associated with incident coronary heart disease by race. ORs and 95% CIs for per SD increase in log-transformed metabolite levels were obtained using conditional logistic regression in each racial group, adjusting for age, education, income, cigarette smoking, alcohol consumption, physical activity, diet quality score, and BMI (model 2).

We further evaluated whether those metabolite-CHD associations differ by participant demographics, lifestyles, metabolic disease history, family history of CHD, and follow-up time through stratified analyses and interaction testing. Several metabolites showed numerically stronger associations among Black than White or Asian participants (Fig 5), including 4-hydroxyphenylacetate (OR 1.37, 95% CI [1.13, 1.65]; *p* < 0.001) and phenylacetyl-L-glutamine (OR 1.32, 95% CI [1.10, 1.58]; *p* = 0.003) among Black participants (*p*-interaction <0.05). Meanwhile, 1-methyl-4-imidazoleacetate was significant only among White participants (OR 1.73, 95% CI [1.16, 2.58]; *p* = 0.007; *p*-interaction <0.05). TMAO was significant only among American participants of Black (OR 1.39, 95% CI [1.15, 1.68]; *p* = 0.001) and White race (OR 1.45, 95% CI [1.10, 1.92]; *p* = 0.008). Other potential effect modifications include 4-hydroxyhippuric acid by age group and taurine and 3-hydroxy-2-ethylpropionate by obesity status. Trans-4-hydroxyproline, phenylacetyl-L-glutamine, p-cresol-sulfate, and 3-phenylpropionate showed stronger associations for CHD diagnosed within two years after baseline (*p*-values for interaction <0.05, but none were significant after FDR correction). No significant interactions were found for sex, hypertension status, diabetes status, dyslipidemia status, physical activity, diet quality, or family history of CHD. Detailed results, including ORs (95% CI) in each subgroup, relative ORs (95% CI) between subgroups, and *p*-values and FDR-*p* for interaction, are shown in S5 Table.

## Discussion

In this multi-stage metabolomic study involving five prospective cohorts, we identified and validated circulating gut microbiota-related metabolites linked to incident CHD. Major strengths include demographically and geographically diverse participants and a rigorous design with discovery, in-silico validation, and quantitative validation. Most of the findings were consistent across gender and racial groups and participants’ metabolic health statuses, suggesting broad generalizability of those metabolite–CHD associations, despite some metabolite concentrations may vary across populations. This study presents new epidemiological evidence supporting the role of gut microbiota in CHD etiology and highlights microbial metabolites and pathways as potential novel biomarkers or therapeutic targets for future mechanistic and interventional studies.

Our results, particularly from quantitative validation, confirmed the role of microbial amino acid metabolites in cardiometabolic diseases. Notably, microbial metabolites of AroAA (phenylalanine, tyrosine, tryptophan) were linked to CVD risk in recent large studies, including the MetaCardis consortium of 1,241 European adults [14], Cleveland Clinic GeneBank and LipidCardio study of 4,000 US and 833 European adults undergoing elective coronary angiography [34], and 7,897 US and European adults from 6 cohorts, including ARIC, in the COnsortium of METabolomics Studies (COMETS) [13]. Specifically, phenylalanine metabolites hydroxyphenylacetate and phenylacetyl-L-glutamine were associated with increased risk of CHD or major adverse cardiovascular events (MACE) in all those studies. Moreover, effect sizes were similar: the HR for 4-hydroxyphenylacetate was 1.24 in COMETS (OR 1.19, 95% CI [1.06, 1.33] in ours); the HR for phenylacetyl-L-glutamine was 1.12 in COMETS and 1.17 among 4,241 Swedish adults from the Malmö cohorts [35] (OR 1.14, 95% CI [1.02, 1.27] in ours). Among tyrosine metabolites, 4-hydroxyphenylpyruvate was significant among participants with dyslipidemia in discovery and in-silico validation, but failed quantitative validation. However, 4-hydroxyhippuric acid, a tyrosine pathway and benzoate metabolite, was significant in the quantitative stage (OR 1.20, 95% CI [1.06, 1.36]), even after adjustment for metabolic disease history), but it was not measured in the discovery stage. We added 4-hydroxyhippuric acid to the quantitative assay because of its association with incident MI/MACE in recent studies [34] and confirmed this. Conversely, tryptophan metabolite indolepropionate was associated with lower CHD risk in discovery and quantitative stages, although it was insignificant in ARIC and unavailable in MESA for in-silico validation. Its association attenuated after adjusting for prevalent diabetes, with an OR of 0.89 (95% CI [0.80, 0.99]) in Model 2 and 0.92 (95% CI [0.82, 1.03]) in Model 3, consistent with evidence on its major role in glucose metabolism and diabetes prevention [36–38].

Besides AroAA metabolites, our study identified other amino acid metabolites, including imidazole-propionate (from histidine) and 3-hydroxy-2-ethylpropionate (from isoleucine). The EPIC-Norfolk cohort first reported circulating imidazole-propionate linked to incident CHD and other diseases (heart failure, renal disease), adjusted for age and sex [12]. Our study confirmed and extended its association with incident CHD in a diverse population, which remained significant after adjustment for sociodemographic and lifestyle factors across all stages: discovery OR 1.35 (95% CI [1.21, 1.51]), in-silico validation HR 1.14 (95% CI [1.02, 1.27]), and targeted validation OR 1.26 (95% CI [1.11, 1.42]). 3-hydroxy-2-ethylpropionate (also named 2-ethylhydracrylic acid) indicates isoleucine catabolism defects [39]. Despite substantial evidence linking circulating branched-chain amino acids (BCAAs: valine, leucine, isoleucine) with cardiometabolic diseases [40–42], to our knowledge, no studies have reported elevated circulating 3-hydroxy-2-ethylpropionate with incident CHD. Its precursor, isoleucine, was also elevated in baseline plasma of incident CHD cases (Fig 2). Together, those findings support that impaired BCAA catabolism contributes to CHD development.

Our study also linked microbial metabolites from the lipid pathway to incident CHD, including TMAO (from choline, phosphatidylcholine, and l-carnitine), 3-hydroxybutyrate (from fatty acids and ketogenic amino acids), and sphingomyelin among participants with dyslipidemia. TMAO exemplifies how gut microbial metabolism of dietary intakes (e.g., egg and red meat) affects CVD risk and prognosis. [43,44] Our previous cross-sectional analysis of >32,000 adults from the US, Europe, and Asia reported that elevated circulating TMAO correlated with high animal food intakes and CVD risk factors, including impaired renal function and glycemic control [45]. Our current prospective analysis confirmed its link with incident CHD (Fig 3, OR~1.20), particularly among Americans (Fig 4, OR~1.40). On the other hand, the role of ketone bodies in cardiometabolic health is increasingly discussed but remains controversial. We observed a positive association between circulating 3-hydroxybutyrate (the primary ketone body) and incident CHD across all stages (OR 1.24, 95% CI [1.10, 1.40] in Model 3 of quantitative stage), particularly among individuals without obesity (OR 1.36, 95% CI [1.14, 1.63]). This finding seemed contradictory to emerging research on ketogenic diets or supplements to improve cardiometabolic health [46]. Among free-living individuals without intervention, elevated circulating ketones are more likely due to decreased ketone oxidation rather than increased ketogenesis. Nevertheless, given our significant positive association and inconsistent findings from clinical trials on ketogenic diets/supplements, further research is warranted to clarify the role of ketone bodies/signaling in CVD prevention.

The major strengths of our study include a diverse population, a multi-stage design, and quantitative validation. Analyses by race and metabolic diseases indicated that most metabolite-CHD associations were consistent across participant groups. Quantitative assay revealed that concentrations of many metabolites were comparable between Black and White Americans but differed between US and Asian adults. We presented detailed results, including metabolite IDs, concentrations, and subgroup associations, in the Supplementary files to inform future research. Meanwhile, this is one of the first comprehensive studies to identify and validate circulating microbiota-related metabolites with incident CHD among Black/African Americans, including 4-hydroxyphenylacetate, phenylacetyl-L-glutamine, imidazole-propionate, 3-hydroxy-2-ethylpropionate, *trans-*4-hydroxyproline, and TMAO (all ORs >1.25 in the quantitative stage). The associations between circulating microbial metabolites and health outcomes may vary among individuals with different ethnicities and dietary/lifestyle habits, or from different regions (thus different environmental exposures). For example, TMAO was significant among Americans but not among Chinese, possibly due to varied dietary sources of TMAO precursors (e.g., red meat versus fish) [45,47]. We also found stronger associations of 3-hydroxy-2-ethylpropionate and 3-hydroxybutyrate among individuals without obesity, suggesting impaired BCAA and ketone body catabolism may underlie “metabolically unhealthy normal weight” phenotype and CVD risk. Further research on these pathways may be fruitful. Yet, since most metabolites showed no significant p-interaction by race and none of the p-interactions were significant after FDR correction, larger future studies with diverse populations are needed to evaluate if certain metabolites are population-specific or general CVD risk factors.

Several limitations of our current study merit discussion. First, we were unable to validate all significant metabolites in the discovery or in-silico validation. Due to the differences in metabolomics assays, 12 significant metabolites from discovery were unavailable for in-silico validation, including butyrate, l-urobilinogen, and an unnamed metabolite (X-12411, associated with incident CHD in EPIC-Norfolk). Also, given the limited coverage of our quantitative assay, 16 metabolites that passed in-silico validation could not be measured simultaneously, such as dihomo-linolenate (20:3n3 or n6) and sphingomyelin (d18:1/21:0, d17:1/22:0, d16:1/23:0). Specific assays for these metabolites are needed to verify the findings. Second, although we broadly adjusted for potential confounders, including sociodemographics, lifestyle factors (smoking, diet quality, alcohol intake, and physical activity), and history of metabolic diseases (diagnosis or medication use), residual confounding may still exist due to inadequate adjustment or unmeasured confounders. For example, kidney function may confound or mediate the metabolite-CHD association; however, we could not include it in our analysis due to a lack of data in all primary cohorts (SCCS and SWHS/SMHS). Also, although we adjusted for overall diet quality, some metabolites, particularly phenolic compounds (e.g., 4-hydroxyphenylacetate and 3-phenylpropionate), can originate from both gut microbial metabolism of dietary polyphenols and direct dietary intake. Third, despite validation in independent samples, spurious associations remain possible from multiple comparisons; thus, the findings warrant further replication. In addition, given the observational nature of this study, the causal role of highlighted microbial metabolites in CHD etiology and therapeutics requires investigation. The associations of some metabolites appeared stronger for CHD cases diagnosed within the first two years of follow-up, which might partly reflect preclinical CHD. Emerging studies manipulating the gut microbiome or metabolites show promise for CVD prevention and treatment [4,48]. Finally, none of the cohorts collected stool samples at the time of blood draw, so we could not link gut microbiome or fecal metabolites with blood metabolites. However, even with stool samples, metagenomics reveals microbiome composition and functional potential, not activity, and fecal metabolites can exhibit substantial within-person variations. Also, although the primary source of circulating microbial metabolites is gut microbiota, microbiota from other sites, e.g., oral microbiota, may also contribute. On the other hand, circulating metabolites reflect functional outputs of the microbiota and microbiota-host interactions most relevant to the host cardiovascular system, which also represent the currently most feasible way to study gut microbial metabolism with incident CHD in existing large cohorts. Future studies collecting multiple types of biospecimens, following participants for incident CVD, and applying multi-omics will further shed light on the role of microbiota-host interactions in CVD development and prevention.

In conclusion, we systematically examined the associations between circulating gut microbiota-related metabolites and incident CHD among demographically and geographically diverse participants. We identified and validated microbial metabolites of amino acids, lipids, and xenobiotics linked to incident CHD. Our findings support the important role of gut microbiota and microbial metabolism in CHD etiology and highlight promising microbial metabolites and pathways that may serve as novel biomarkers or therapeutic targets for future mechanistic and interventional studies.

## Supporting information

S1 Text Supplemental Protocol and Figs A and B. (DOCX)

S1 Table Baseline characteristics of participants in the in-silico validation. (XLSX)

S2 Table Associations between circulating gut microbiota-related metabolites and incident coronary heart disease in the discovery stage. (XLSX)

S3 Table In-silico validation of associations between circulating gut microbiota-related metabolites and incident coronary heart disease in ARIC and MESA. (XLSX)

S4 Table Plasma concentrations of circulating gut microbiota-related metabolites in CHD cases and non-CHD controls in the targeted validation stage. (XLSX)

S5 Table Stratified analyses of associations between circulating gut microbiota-related metabolites and incident coronary heart disease in the targeted validation stage. (XLSX)

S1 STROBE Checklist STROBE Statement—checklist of items that should be included in reports of observational studies. Licensed under CC BY 4.0. Checklist available from https://www.strobe-statement.org/checklists/.
(DOCX)

## Abbreviations

ARIC: Atherosclerosis Risk in Communities Study
AroAA: aromatic amino acids
BCAA: branched-chain amino acids
BMI: body mass index
CHD: coronary heart disease
CIs: confidence intervals
CMS: Centers for Medicare and Medicaid Services
COMETS: COnsortium of METabolomics Studies
CVD: cardiovascular disease
FDR: false discovery rate
MESA: Multi-Ethnic Study of Atherosclerosis
MI: myocardial infarction
ORs: odds ratios
SCCS: Southern Community Cohort Study
SD: standard deviation
STROBE: Strengthening the Reporting of Observational Studies in Epidemiology
SWHS/SMHS: Shanghai Women’s Health Study and Shanghai Men’s Health Study
TMAO: trimethylamine N-oxide
