# Supplementary material for: Circulating gut microbial metabolites and risk of coronary heart disease: A prospective multi-stage metabolomics study
Source: PLoS Med. 2026 Mar 17;23(3):e1004750. doi: 10.1371/journal.pmed.1004750 (PMC12994840; doi:10.1371/journal.pmed.1004750)
Supplement: S1 Text — (DOCX) [file pmed.1004750.s001.docx]

### **Supplemental Protocol and Figs A-B**

### **S Protocol**

#### **Parent cohorts**

The Southern Community Cohort Study (SCCS)^1^ was established to investigate race/ethnicity and socioeconomic status-related health disparities in the US. From 2002–2009, 84,735 men and women (40–79 years) were recruited from 12 southern US states, mostly from community health centers that serve low-income, uninsured populations. Approximately 65% of the SCCS participants were self-reported Black/African American, and >80% reported annual household income <$25,000. A structured questionnaire was administered to collect baseline information on sociodemographics, disease history, medication use, and lifestyles, including habitual diet. Among 73,500 participants recruited from community health centers, 39,500 donated a blood sample, which was transported at 4°C to Vanderbilt University Medical Center Molecular Epidemiology Core Laboratory and stored at -80°C. Death and chronic disease outcomes are identified via follow-up surveys and linkages to the National Death Index and Centers for Medicare and Medicaid Services (CMS) Files. Nonfatal CHD cases were identified based on ICD and CPT codes using an algorithm adapted from other studies and validated in the SCCS, including acute myocardial infarction (MI; ICD-10 code: I21), coronary revascularization (ICD-10-PCS codes: 021xxxx or 027xxxx; or CPT codes: 92980-92996 or 33510-33536), and other CHD (ICD-10 codes: I24.8, I24.9, I25.1, or I25.6-I25.9). Meanwhile, CHD deaths were identified through the underlying cause of death (ICD-10 codes: I20-I25). The SCCS was approved by the Institutional Review Boards of the Vanderbilt University Medical Center and Meharry Medical College (#010345), with informed consent obtained from all participants. The present study was approved by the SCCS Data and Biospecimen Use Committee.

The Shanghai Women’s Health Study (SWHS)^2^ and Shanghai Men’s Health Study (SMHS)^3^ are prospective cohorts of 74,940 women and 61,480 men (40-74 years) recruited in Shanghai, China from 1996–2000 and 2002–2006, respectively. A baseline interview was conducted by trained staff to administer questionnaires, measure anthropometrics, and collect biospecimens. Blood samples were collected from ~75% of participants. All samples were transported at 4°C to the Shanghai Cancer Institute, processed within six hours of collection, and stored at -80°C. Death and chronic disease outcomes were identified via follow-up home visits and linkages to the Shanghai vital statistics and medical records. Incident CHD cases were identified by self-reported physician diagnosis and confirmed by medical records. The SWMHS was approved by the Institutional Review Boards of the Vanderbilt University Medical Center and Shanghai Cancer Institute (#000340 and #000598), with informed consent obtained from all participants. The present study was approved by the SWHS/SMHS Data and Biospecimen Use Committee.

The Atherosclerosis Risk in Communities (ARIC) study^4,5^ is an ongoing prospective cohort study that started in 1987-1989 with 10 completed visits until 2023. At baseline, it recruited 15,792 mostly Black and White individuals (45–65 years) across four communities in the US, including Forsyth County, North Carolina; Minneapolis, Minnesota; Washington County, Maryland; and Jackson, Mississippi. Data collection consisted of in-person examinations and phone interviews. Follow-up assessments include visit 2 in 1990-1992, visit 3 in 1993-1995, visit 4 in 1996-1998, visit 5 in 2011-2013, visit 6 in 2016-2017, visit 7 in 2018-2019, visit 8 in 2020, visit 9 in 2021-2022, and visit 10 in 2023. Incident CHD is defined as definite or probable MI, fatal CHD, and if participants had undergone any cardiac procedures or ECG MI prior to December 31st, 2018. The cases were ascertained by annual phone calls and medical records.

The Multi-Ethnic Study of Atherosclerosis (MESA)^6^ is a prospective cohort of 6,814 adults (45–84 years) recruited from six centers across the US in 2000–2002 without known clinical cardiovascular disease. Around 38% of participants are self-described White, 28% Black, 22% Hispanic/Latino, and 12% Chinese. The baseline exams included information on a wide range of CVD risk factors, including lifestyle and biomarkers such as blood lipids and glucose. Participants were followed for incident CVD at yearly intervals from the baseline exam. In addition to follow-up study visits (exams 1-7), a telephone interviewer contacted each participant at least yearly to inquire about all interim hospital admissions, cardiovascular diagnoses, anword deaths. Copies of death certificates and medical records for hospitalizations and cardiovascular diagnoses were obtained; alongside next-of-kin interviews where appropriate. Two physicians independently reviewed all medical records for endpoint classification and assignment of incidence dates. In the rare case of disagreements, differences are adjudicated and moved to a full committee, if resolution is not obtained.

#### **Metabolite profiling**

For the discovery stage, untargeted metabolite profiling was performed using ultra-high-performance liquid chromatography (UHPLC) coupled with tandem mass spectrometry (MS) by Metabolon Inc. (Morrisville, NC, USA). The general assay protocol has been published^7^. Briefly, plasma samples were extracted with methanol and split into four aliquots for analysis by UHPLC-MS/MS in positive and negative ion modes using a combination of reverse phase and HILIC chromatography methods. Equipment included a Waters ACQUITY UHPLC, a Thermo Scientific Q-Exactive high resolution/accurate mass spectrometer interfaced with a heated electrospray ionization source, and an Orbitrap mass analyzer operated at 35,000 mass resolution. The sample extract was dried and reconstituted in solvents compatible with each of the four methods. Each reconstitution solvent contained a series of standards at fixed concentrations to ensure injection and chromatographic consistency. One aliquot was analyzed using acidic positive ion conditions, chromatographically optimized for hydrophilic compounds. In this method, the extract was gradient eluted from a C18 column (Waters UPLC BEH C18-2.1x100 mm, 1.7 µm) using water and methanol, containing 0.05% perfluoropentanoic acid (PFPA) and 0.1% formic acid (FA). Another aliquot was also analyzed using acidic positive ion conditions but chromatographically optimized for hydrophobic compounds. In this method, the extract was gradient eluted from the same C18 column using methanol, acetonitrile, water, 0.05% PFPA and 0.01% FA and was operated at an overall higher organic content. Another aliquot was analyzed using basic negative ion optimized conditions using a separate dedicated C18 column. The basic extracts were gradient eluted from the column using methanol and water, with 6.5mM Ammonium Bicarbonate at pH 8. The fourth aliquot was analyzed via negative ionization following elution from a HILIC column (Waters UPLC BEH Amide 2.1x150 mm, 1.7 µm) using a gradient consisting of water and acetonitrile with 10mM Ammonium Formate, pH 10.8. The MS analysis alternated between MS and data-dependent MSn scans using dynamic exclusion. The scan range covered 70-1000 m/z. Raw data was extracted, peak-identified, and QC processed. Metabolites were identified by automated comparison of mass spectra features to a reference library of >4,000 authenticated standard compounds, followed by visual inspection for QC. Peaks were quantified using area-under-the-curve. A total of 1503 metabolites were detected (1229 named and 274 unnamed) in the discovery stage samples of SCCS and SWHS/SMHS, with the majority (>80%) being annotated based on standard compounds. In ARIC, baseline serum samples were also analyzed by Metabolon Inc. using the same methods, which detected 787 metabolites.

The MESA dataset contained metabolites measured by both LC-MS and NMR. Each sample was mixed with four parts of 4 °C isopropanol, incubated at 4 °C for 2 h in a plate shaker at 1,400 rpm, centrifuged, and the supernatant aliquoted into a 96-well plate. LC-MS data acquisition was described previously.^8^ LC-MS data was acquired on Acquity UPLC systems coupled to Xevo G2-S ToF mass spectrometers (Waters Corporation, Milford, MA, US), using three assays: lipid profiling by reversed-phase chromatography in both positive and negative ion mode, and small molecule profiling by hydrophilic interaction liquid chromatography in positive ion mode. LC-MS metabolite annotation was performed by matching m/z values to the metabolites in the discovery dataset. Some of the metabolomic features were annotated by matching accurate mass fragmentation data to reference spectra from online databases (LIPID MAPS, Metlin, and HMDB) and literature. When commercial reference materials (Avanti Polar Lipids, Sigma Aldrich, Cayman Scientific) were available, they were used for definitive molecular identification by directly matching chromatographic and spectral properties, including accurate mass, MS/MS spectra, and isotopic distribution. NMR data acquisition was also described previously.^9,10^ Briefly, a spectrum using the Carr-Purcell-Meiboom-Gill sequence was obtained for each sample, allowing the detection of small molecular species. TOPSPIN 3.1 (Bruker Biospin, Rheinstetten, Germany) software was used for spectral processing. Spectra were automatically phased and baseline corrected, and the chemical shifts calibrated to the glucose signal at 5.233 ppm. NMR metabolite annotation was done as previously reported.^9,10^ After peak areas were automatically quantified, the chemical shift was compared with available in-house and publicly available databases, and when chemical standards were available, spiking experiments were performed to validate the identity of the metabolite. When there were overlapping metabolites in the LC-MS and NMR datasets, metabolites from the NMR dataset were used. All metabolite data were log-scaled and z-scored prior to statistical analyses.

For the targeted validation stage, 16 metabolites were quantified using LC-MS/MS by Metabolon Inc. Two sample sets for each batch (Reverse Phase and HILIC) were prepared from a single plasma sample. Plasma samples were spiked with isotopically labelled internal standard and subjected to protein precipitation with an acidified organic solution. An aliquot was injected onto an Agilent 1290/Sciex Triple Quad 6500+ LC-MS/MS system equipped with either an Agilent Zorbax SB-C18 RRHD for the reverse phase method or a Waters Acquity Premier BEH Amide column for the HILIC method. The mass spectrometer was operated in both positive and negative modes using heated electrospray ionization. The peak area of the individual analyte product ions was measured against the peak area of the product ions of the corresponding internal standards. Quantitation was performed using a weighted linear least squares regression analysis generated from fortified calibration standards prepared concurrently with study samples. LC-MS/MS raw data are collected using Sciex Analyst software and processed using Sciex OS-MQ software. Data reduction is performed using Microsoft Excel for Microsoft 365 MSO. Sample analysis was conducted in a 96-well plate format containing two calibration curves and six QC samples (three levels with two at each level). Accuracy was evaluated using the QC replicates in the sample runs. The average accuracy for QC at all levels for all 16 analytes was >90% except for 1-methyl-4-imidazoleacetate (average accuracy for QCs = 88%). The median coefficient of variation for those 16 metabolites among our study QC samples was 3.6% (25^th^-75^th^ percentile: 1.6%-6.4%).

**Reference standards for targeted quantitative assay**

| **Analyte Name** | **Manufacturer** | **Cat #** | **Lot #** |
| --- | --- | --- | --- |
| 1-methyl-4-imidazoleacetate | Cayman | 18815 | 0515697 |
| imidazole propionate | Sigma-Aldrich | 77951 | BCCH4693 |
| 3-hydroxy-2-ethylpropionate | SantaCruz | sc-397063 | L0722 |
| alpha-ketobutyrate | Sigma-Aldrich | K401 | BCBW9441 |
| 4-hydroxyphenylacetate | Sigma-Aldrich | H50004 | BCCG2600 |
| 3-indolepropionate | Sigma-Aldrich | 220027 | 0001431484 |
| 4-hydroxyphenylpyruvate | Sigma-Aldrich | 114286 | BCCG7346 |
| 3-hydroxybutyrate | Sigma-Aldrich | 54920 | BCCC5741 |
| 3-phenylpropionate | Sigma-Aldrich | 80725 | BCCF4377 |
| 2,6-dihydroxybenzoic acid | Sigma-Aldrich | D109606 | MKCQ4053 |
| trans-4-hydroxyproline | Sigma-Aldrich | 41875 | BCCD0807 |
| trimethylamine N-oxide | Sigma-Aldrich | 317594 | SHBL0520 |
| taurine | Sigma-Aldrich | T0625 | BCCC3480 |
| p-Cresol Sulfate | Cayman | 29504 | 634005 |
| 4-Hydroxyhippuric acid | Cayman | 30115 | 0582183-16 |
| Phenylacetyl-L-Glutamine | Sigma-Aldrich | SMB00962 | MKCS8960 |
| 1-methyl-d3-1H-imidazol-4-yl acetate | BOC Sciences | 122380-33-0 | B23TS01041 |
| Imidazole propionate-d3 | Metabolon | N/A | 242-36-B2 |
| (4-hydroxyphenyl-2,3,5,6-d4) acetate-2,2-d2 | CDN | D-7842 | AC-307 |
| 2-ketobuytrate-4-13C-3,3-d2 | Sigma-Aldrich | 589276 | MBBD0456 |
| Indole-3-propionic-2,2-d2 Acid | CDN | D-7686 | I-408 |
| 4-hydroxyphenylpyruvate 13C9 | SIAL | 7491 | BCCH6282 |
| Sodium (+/-)-3-Hydroxybutyrate-3,4,4,4-d4 | CDN | D-6088 | R-228 |
| 3-phenyl-d5-propionate | CDN Isotopes | D-5846 | K-178 |
| trans-4-hydroxyproline-d3 | CDN | D-7186 | AC-283 |
| trimethylamine N-oxide, d9 | CDN | PR-30947 | DLM-4779-0 |
| taurine-d4 | CDN | D-1971 | CD-267 |
| p-Cresol-d7 | Santa Cruz | Sc-474750 | L1120 |
| 2-Hydroxyhippuric acid-13C2,15N | TRC | H942887 | 6-AQL-42-1 |
| Phenylacetyl-d5-L-glutamine | CDN | D-6900 | JK-220 |

**Calibration standards and ranges**

| **Analyte** | **Target Standard Concentration (ng/mL)** | | | | | | | |
| --- | --- | --- | --- | --- | --- | --- | --- | --- |
|  | **A** | **B** | **C** | **D** | **E** | **F** | **G** | **H** |
| 1-methyl-4-imidazoleacetate | 0.128 | 0.256 | 0.640 | 1.60 | 3.20 | 9.60 | 13.0 | 16.0 |
| imidazole propionate | 0.400 | 0.800 | 2.00 | 5.00 | 10.0 | 30.0 | 40.0 | 50.0 |
| 3-hydroxy-2-ethylpropionate | 4.00 | 8.00 | 20.0 | 50.0 | 100 | 300 | 400 | 500 |
| alpha-ketobutyrate | 80.0 | 160 | 400 | 1000 | 2000 | 6000 | 8000 | 10000 |
| 4-hydroxyphenylacetate | 40.0 | 80.0 | 200 | 500 | 1000 | 3000 | 4000 | 5000 |
| indolepropionate | 12.8 | 25.6 | 64.0 | 160 | 320 | 960 | 1280 | 1600 |
| 4-hydroxyphenylpyruvate | 32.0 | 64.0 | 160 | 400 | 800 | 2400 | 3200 | 4000 |
| 3-hydroxybutyrate (BHBA) | 1000 | 2000 | 5000 | 12500 | 25000 | 75000 | 100000 | 125000 |
| 3-phenylpropionate | 1000 | 2000 | 5000 | 12500 | 25000 | 75000 | 100000 | 125000 |
| 2,6-dihydroxybenzoic acid | 1.28 | 2.56 | 6.40 | 16.0 | 32.0 | 96.0 | 128 | 160 |
| trans-4-hydroxyproline | 64.0 | 128 | 320 | 800 | 1600 | 4800 | 6400 | 8000 |
| trimethylamine N-oxide | 48.0 | 96.0 | 240 | 600 | 1200 | 3600 | 4800 | 6000 |
| taurine | 128 | 256 | 640 | 1600 | 3200 | 9600 | 12800 | 16000 |
| p-Cresol Sulfate | 32.0 | 64.0 | 160 | 400 | 800 | 2400 | 6400 | 8000 |
| 4-Hydroxyhippuric acid | 1.00 | 2.00 | 5.00 | 12.5 | 25.0 | 75.0 | 200 | 250 |
| Phenylacetyl-L-glutamine | 8.00 | 16.0 | 40.0 | 100 | 200 | 600 | 1600 | 2000 |

**QC performance for the targeted quantitative assay**

| **Analyte** | **QC Low** | | **Dilution QC (QC Med)** | | **QC Medium** | | **QC High** | |
| --- | --- | --- | --- | --- | --- | --- | --- | --- |
|  | **Average Conc (ng/mL)** | **Average Accuracy (%)** | **Average Conc (ng/mL)** | **Average Conc (ng/mL)** | **Average Conc (ng/mL)** | **Average Accuracy (%)** | **Average Conc (ng/mL)** | **Average Accuracy (%)** |
| 1-methyl-4-imidazoleacetate | 0.359 | 86.7 | 2.02 | 90.9 | 1.88 | 84.5 | 9.71 | 87.5 |
| 2,6-dihydroxybenzoic acid | 3.23 | 94.5 | 16.5 | 98.3 | 15.9 | 94.5 | 130 | 93.3 |
| 3-hydroxy-2-ethylpropionate | 9.70 | 106 | 85.5 | 106 | 75.1 | 93.5 | 315 | 94.0 |
| 3-phenylpropionate | 3037 | 98.9 | 18050 | 97.5 | 18210 | 98.3 | 85473 | 94.8 |
| 4-hydroxyphenylacetate | 113 | 96.2 | 583 | 90.4 | 600 | 93.1 | 3232 | 94.1 |
| 4-hydroxyphenylpyruvate | 547 | 103 | 3210 | 110 | 2999 | 102 | 14305 | 101 |
| imidazole propionate | 1.02 | 95.8 | 5.12 | 89.8 | 5.46 | 95.7 | 31.4 | 91.9 |
| indolepropionate | 31.8 | 96.1 | 207 | 92.4 | 210 | 94.0 | 1025 | 91.4 |
| 3-hydroxybutyrate (BHBA) | 2969 | 91.3 | NA | NA | 18200 | 94.0 | 86690 | 92.8 |
| alpha-ketobutyrate | 207 | 96 | 1660 | 116 | 1509 | 105.0 | 11374 | 112 |
| taurine | 370 | 94.3 | NA | NA | 1888 | 91.8 | 10337 | 91.1 |
| trans-4-hydroxyproline | 173 | 96.8 | NA | NA | 982 | 95.5 | 4856 | 92.7 |
| trimethylamine N-oxide | 140 | 96.7 | NA | NA | 815 | 94.4 | 4281 | 93.6 |
| 4-Hydroxyhippuric acid | 1.21 | 95.3 | NA | NA | 6.09 | 100 | 32.0 | 95.0 |
| p-Cresol Sulfate | 67.4 | 98.6 | NA | NA | 387 | 101 | 2262 | 99.1 |
| Phenylacetyl-L-glutamine | 12.1 | 98.6 | NA | NA | 68.8 | 101 | 418 | 97.5 |

**Supplemental references**

1. Signorello LB, Hargreaves MK, Blot WJ. The Southern Community Cohort Study: investigating health disparities. *J Health Care Poor Underserved.* 2010;21(1 Suppl):26-37.

2. Zheng W, Chow WH, Yang G, et al. The Shanghai Women's Health Study: rationale, study design, and baseline characteristics. *Am J Epidemiol.* 2005;162(11):1123-1131.

3. Shu XO, Li H, Yang G, et al. Cohort Profile: The Shanghai Men's Health Study. *Int J Epidemiol.* 2015;44(3):810-818.

4. The Atherosclerosis Risk in Communities (ARIC) Study: design and objectives. The ARIC investigators. *Am J Epidemiol.* 1989;129(4):687-702.

5. Wright JD, Folsom AR, Coresh J, et al. The ARIC (Atherosclerosis Risk In Communities) Study: JACC Focus Seminar 3/8. *J Am Coll Cardiol.* 2021;77(23):2939-2959.

6. Bild DE, Bluemke DA, Burke GL, et al. Multi-Ethnic Study of Atherosclerosis: objectives and design. *Am J Epidemiol.* 2002;156(9):871-881.

7. Evans AM, DeHaven CD, Barrett T, Mitchell M, Milgram E. Integrated, nontargeted ultrahigh performance liquid chromatography/electrospray ionization tandem mass spectrometry platform for the identification and relative quantification of the small-molecule complement of biological systems. *Anal Chem.* 2009;81(16):6656-6667.

8. Lewis MR, Pearce JT, Spagou K, et al. Development and Application of Ultra-Performance Liquid Chromatography-TOF MS for Precision Large Scale Urinary Metabolic Phenotyping. *Anal Chem.* 2016;88(18):9004-9013.

9. Dona AC, Jimenez B, Schafer H, et al. Precision high-throughput proton NMR spectroscopy of human urine, serum, and plasma for large-scale metabolic phenotyping. *Anal Chem.* 2014;86(19):9887-9894.

10. Tzoulaki I, Castagne R, Boulange CL, et al. Serum metabolic signatures of coronary and carotid atherosclerosis and subsequent cardiovascular disease. *Eur Heart J.* 2019;40(34):2883-2896.


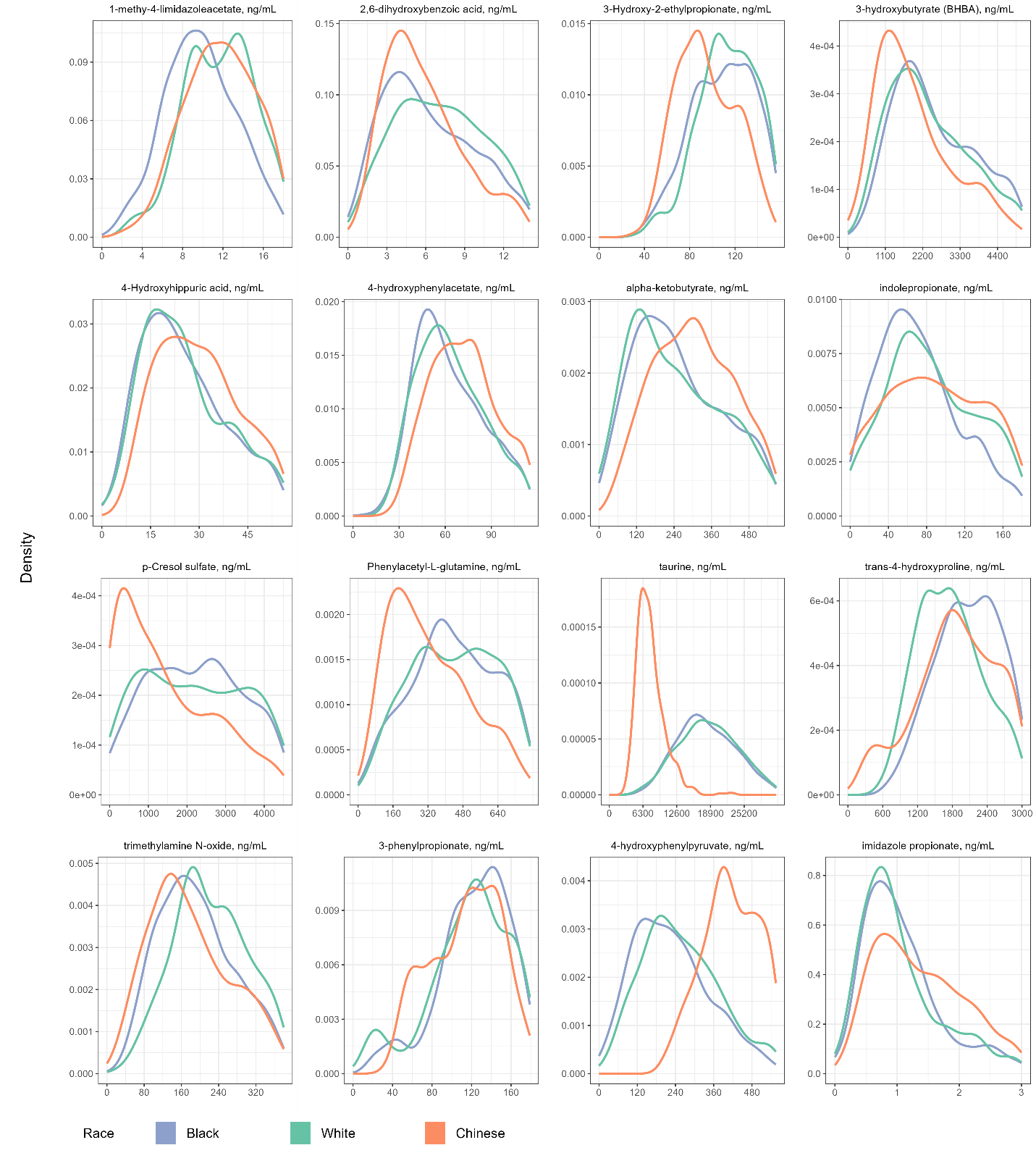


**Fig A. Concentrations of 16 metabolites by race in targeted validation samples.** Density plots show the distributions of plasma concentrations of 16 metabolites among Black, White, and Chinese participants. Each panel represents a metabolite measured by targeted liquid chromatography-mass spectrometry. The x-axis represents metabolite concentration (ng/mL), and the y-axis represents the relative frequency distribution (density). Colored curves represent different racial groups: Black (blue), White (green), and Chinese (orange).

**
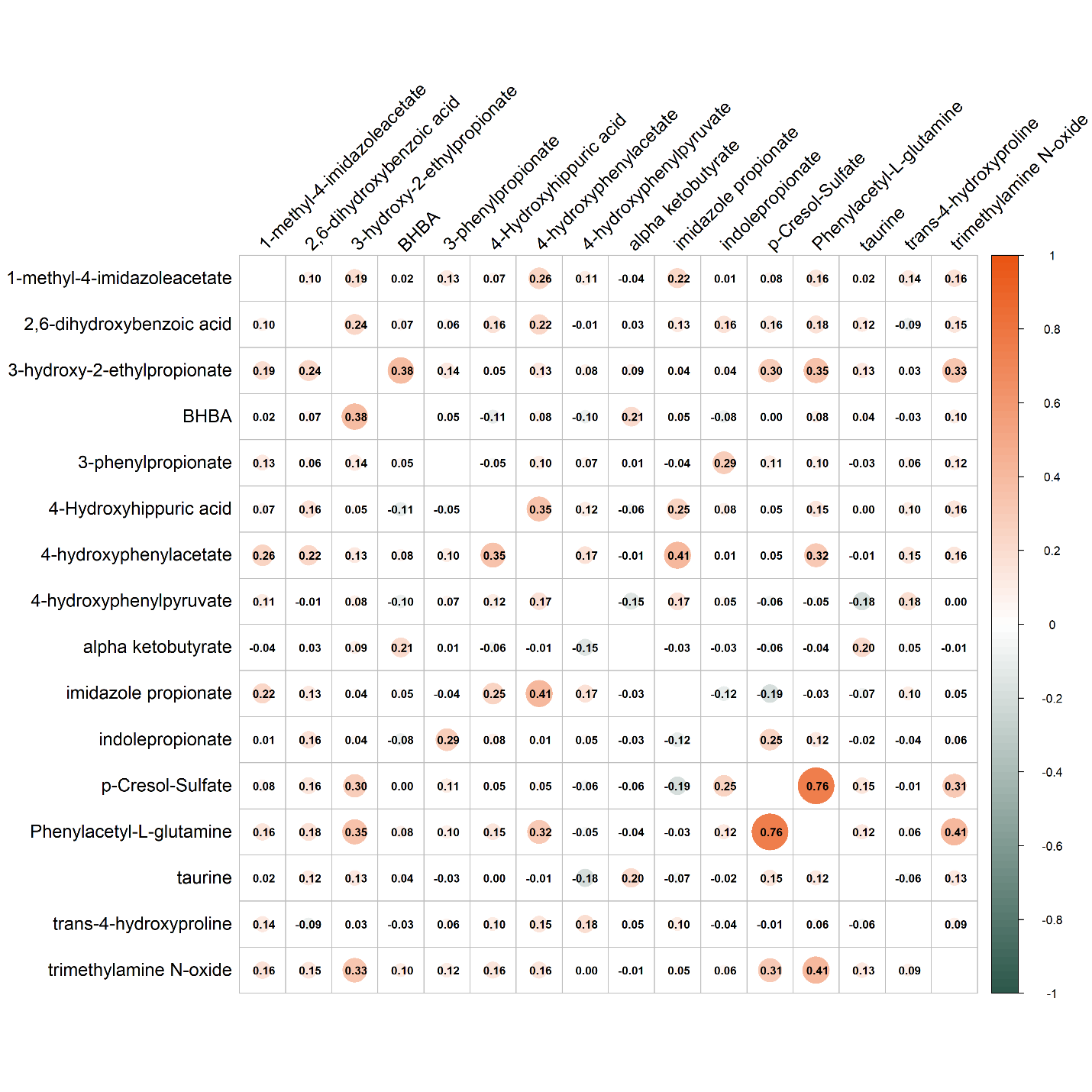
**

**Fig B. Spearman correlation coefficients among 16 metabolites in targeted validation.** Heatmap shows pairwise Spearman partial correlation coefficients between 16 plasma metabolites measured in targeted validation stage, adjusted for age, sex, race, and fasting status. Circle size and color intensity represent the magnitude of correlation: larger circles and darker colors indicate stronger correlations. Green indicates negative correlations; orange indicates positive correlations. Correlation coefficients range from -0.19 to 0.76.
