# Supplementary material for: Circulating gut microbial metabolites and risk of coronary heart disease: A prospective multi-stage metabolomics study
Source: PLoS Med. 2026 Mar 17;23(3):e1004750. doi: 10.1371/journal.pmed.1004750 (PMC12994840; doi:10.1371/journal.pmed.1004750)
Supplement: S1 STROBE Checklist — Licensed under CC BY 4.0. Checklist available from https://www.strobe-statement.org/checklists/. (DOCX) [file pmed.1004750.s007.docx]

STROBE Statement—checklist of items that should be included in reports of observational studies

|  | Item No. | Recommendation | Section and paragraph  No. | Relevant text from manuscript |
| --- | --- | --- | --- | --- |
| **Title and abstract** | 1 | (*a*) Indicate the study’s design with a commonly used term in the title or the abstract | Title | A prospective multi-stage study |
|  |  | (*b*) Provide in the abstract an informative and balanced summary of what was done and what was found | Abstract | Methods and Findings: |
| Introduction | | | |  |
| Background/rationale | 2 | Explain the scientific background and rationale for the investigation being reported | Introduction, paragraphs 1-2 |  |
| Objectives | 3 | State specific objectives, including any prespecified hypotheses | Introduction, paragraph 3 | Hence, we conducted… This study aimed to … |
| Methods | | | |  |
| Study design | 4 | Present key elements of study design early in the paper | Study population and design, paragraph 1 |  |
| Setting | 5 | Describe the setting, locations, and relevant dates, including periods of recruitment, exposure, follow-up, and data collection | Study population and design, paragraph 1 |  |
| Participants | 6 | (*a*) *Cohort study*—Give the eligibility criteria, and the sources and methods of selection of participants. Describe methods of follow-up  *Case-control study*—Give the eligibility criteria, and the sources and methods of case ascertainment and control selection. Give the rationale for the choice of cases and controls  *Cross-sectional study*—Give the eligibility criteria, and the sources and methods of selection of participants | Study population and design, paragraph 2 | For discovery and targeted validation, prospective nested case-control studies were conducted…  The in-silico validation was conducted in ARIC and MESA using a prospective cohort design. |
|  |  | (*b*) *Cohort study*—For matched studies, give matching criteria and number of exposed and unexposed  *Case-control study*—For matched studies, give matching criteria and the number of controls per case | Study population and design, paragraph 2 |  |
| Variables | 7 | Clearly define all outcomes, exposures, predictors, potential confounders, and effect modifiers. Give diagnostic criteria, if applicable | Statistical analysis |  |
| Data sources/ measurement | 8* | For each variable of interest, give sources of data and details of methods of assessment (measurement). Describe comparability of assessment methods if there is more than one group | Metabolites profiling, paragraphs 1-3 |  |
| Bias | 9 | Describe any efforts to address potential sources of bias | Statistical analysis | Model adjustment… Stratified analyses… |
| Study size | 10 | Explain how the study size was arrived at | Study population and design, paragraph 2 |  |

| Quantitative variables | | 11 | | Explain how quantitative variables were handled in the analyses. If applicable, describe which groupings were chosen and why | Metabolites profiling, paragraph 4  Statistical analysis | |  |
| --- | --- | --- | --- | --- | --- | --- | --- |
| Statistical methods | | 12 | | (*a*) Describe all statistical methods, including those used to control for confounding | Statistical analysis | | Model adjustment… |
|  |  |  |  | (*b*) Describe any methods used to examine subgroups and interactions | Statistical analysis | | Stratified analyses… |
|  |  |  |  | (*c*) Explain how missing data were addressed | Metabolites profiling, paragraph 4 | |  |
|  |  |  |  | (*d*) *Cohort study*—If applicable, explain how loss to follow-up was addressed  *Case-control study*—If applicable, explain how matching of cases and controls was addressed  *Cross-sectional study*—If applicable, describe analytical methods taking account of sampling strategy | Study population and design, paragraph 2 | |  |
|  |  |  |  | (*e*) Describe any sensitivity analyses |  | |  |
| Results | | | | | | | |
| Participants | | 13* | | (a) Report numbers of individuals at each stage of study—eg numbers potentially eligible, examined for eligibility, confirmed eligible, included in the study, completing follow-up, and analysed | Baseline characteristics of study participants | |  |
|  |  |  |  | (b) Give reasons for non-participation at each stage |  | |  |
|  |  |  |  | (c) Consider use of a flow diagram | Fig. 1 | |  |
| Descriptive data | | 14* | | (a) Give characteristics of study participants (eg demographic, clinical, social) and information on exposures and potential confounders | Baseline characteristics of study participants | |  |
|  |  |  |  | (b) Indicate number of participants with missing data for each variable of interest | Metabolites profiling, paragraph 4  S1-2 Tables | |  |
|  |  |  |  | (c) *Cohort study*—Summarise follow-up time (eg, average and total amount) | Baseline characteristics of study participants | |  |
| Outcome data | | 15* | | *Cohort study*—Report numbers of outcome events or summary measures over time | Table 1 and S1 Table | |  |
|  |  |  |  | *Case-control study—*Report numbers in each exposure category, or summary measures of exposure |  | |  |
|  |  |  |  | *Cross-sectional study—*Report numbers of outcome events or summary measures |  | |  |
| Main results | | 16 | | (*a*) Give unadjusted estimates and, if applicable, confounder-adjusted estimates and their precision (eg, 95% confidence interval). Make clear which confounders were adjusted for and why they were included | Discovery, In-silico validation, Targeted validation… Figures 2-4 and S2-3 Tables | |  |
|  |  |  |  | (*b*) Report category boundaries when continuous variables were categorized |  | |  |
|  |  |  |  | (*c*) If relevant, consider translating estimates of relative risk into absolute risk for a meaningful time period |  | |  |
| Other analyses | 17 | | Report other analyses done—eg analyses of subgroups and interactions, and sensitivity analyses | | Targeted validation… Fig. 5 and S5 Table |  | |
| Discussion | | | | | | | |
| Key results | 18 | | Summarise key results with reference to study objectives | | Paragraph 1 of the discussion |  | |
| Limitations | 19 | | Discuss limitations of the study, taking into account sources of potential bias or imprecision. Discuss both direction and magnitude of any potential bias | | Strengths and limitations |  | |
| Interpretation | 20 | | Give a cautious overall interpretation of results considering objectives, limitations, multiplicity of analyses, results from similar studies, and other relevant evidence | | Paragraphs 2-4 of the discussion |  | |
| Generalisability | 21 | | Discuss the generalisability (external validity) of the study results | | Paragraph 1 of the discussion |  | |
| Other information | | |  | | | | |
| Funding | 22 | | Give the source of funding and the role of the funders for the present study and, if applicable, for the original study on which the present article is based | | Funding |  | |

*Give information separately for cases and controls in case-control studies and, if applicable, for exposed and unexposed groups in cohort and cross-sectional studies.

**Note:** An Explanation and Elaboration article discusses each checklist item and gives methodological background and published examples of transparent reporting. The STROBE checklist is best used in conjunction with this article (freely available on the Web sites of PLoS Medicine at http://www.plosmedicine.org/, Annals of Internal Medicine at http://www.annals.org/, and Epidemiology at http://www.epidem.com/). Information on the STROBE Initiative is available at www.strobe-statement.org.
